# Amalgam

**Published:** 1848-01

**Authors:** 


*Amalgam.*—So extensively has this article been used for filling teeth—so ample have been the opportunities of almost every practitioner of dental surgery of observing its effects, and so frequently have we expressed our opinion with regard to its suitableness for this purpose, that we had not supposed it necessary to recur to the subject again, and we do so very reluctantly, because of the unpleasantness of feeling that has already resulted from the discussion to which it has, within the last two or three years, given rise; but, as we have recently been asked for our views in relation to it, we will again give them as briefly as possible.

During the last twelve years, we have seen some hundreds, perhaps a thousand or more, of teeth, which had been filled with amalgam, and we do not recollect to have met with a single case in which either some bad effect had not resulted from its use, or the decay in the tooth was not as perfectly arrested as it might have been with gold. It is true, in perhaps one-fourth of the cases the teeth were dead, and had acted as morbid irritants before they were filled, and could not have been prevented from doing this by any treatment, but the diseased action which they excited, had, evidently, in most of these cases, been very greatly aggravated, and would have been in some of them, even had gold been employed. But in many of the teeth which had not lost their vitality, a diseased action had been set up in the gums and alveolo-dental periosteal tissues, subsequently to their having been filled, as a consequence of the presence of the amalgam in the teeth, as was proven by the fact, that, in a number of cases from which it was removed, these parts immediately assumed a healthy condition. We have, also, known it in a number of instances, where several large cavities in the same mouth had been filled with it, to produce salivation, and it always gives to the teeth a disagreeable aspect, often causing them to assume a greenish or black appearance.

We believe the most strenuous advocates for its use, acknowledge it to be a bad material for filling teeth—altogether inferior to gold, and only recommend the employment of it in those cases, where, as, they say, the latter article cannot be used. This, so far as we have been able to learn their sentiments with regard to it, is the position which they assume, and if it can be shown that any tooth that can remain in the mouth without exerting a morbid influence upon the surrounding parts, can be filled with gold, their advocacy of its use, must cease. The affirmative of this question has already been assumed in the proposition made by Dr. Westcott, more than a year ago, to fill any such tooth with gold, provided it can be filled with amalgam, and no one, that we are aware of, has yet accepted the challenge. Until this is done, and it is shown that the Dr. is unable to redeem his pledge, we are bound to maintain that gold may be successfully employed in *all cases* in which it is proper to attempt the operation. Our own experience and observations fully warrant the belief that Dr. W. will be able to redeem his pledge whenever put to the test. At any rate we have yet to see the first case, in which it could not have been done. We have replaced amalgam fillings with gold, in many, very many cases, in which the patients had been informed that it was impossible so fill these teeth with any other material, and not in a ***single instance***, to the best of our recollection, have we failed to do this, after having removed a filling of this sort with that intention. We replaced several a few weeks since for a gentleman from Virginia, who had them put in his teeth, a few days before, by a dentist in New York, who assured him, that gold fillings could not be inserted as the teeth were too much decayed to retain this latter material. The teeth, it is true, were badly decayed, but we succeeded in filling every one of them with gold, but in two of which, we used about eighteen leaves of No. 4, and bestowed upon them eight hours hard labor. We were occupied, altogether, in the operations upon the teeth of this individual about three days.

The only advantage, then, which we can perceive, in the use of amalgam is, that it saves labor, and if it saved teeth as perfectly as does the skilful use of gold, this, it must be confessed, would be a great matter, but as no one pretends to argue that it does, we must contend for the use of the latter, in *all cases*, in preference to the former, and until we shall have more convincing proofs that we are in error, we shall continue to use it. We do not, however, wish to dictate to others the practice which they should pursue, and however much we might desire to persuade such as contend for the use of amalgam only in "certain cases," that they are in error, we would not do it, as they may be as honest in their convictions as we are in ours, without convincing them of the fact. We think we might do this with some, if we could persuade them to bestow from two to four, or, if necessary, six hours labor, upon a gold filling, in a tooth which they have hitherto been in the habit of regarding as incapable of being filled with any other material than amalgam. It is in this way that we have become fully convinced that any tooth susceptible of being securely filled with the last mentioned material, can be filled with gold, if the organ can remain in the mouth with impunity. If this can be done, no matter how great the labor, or large may be the quantity of gold required in the operation, we cannot believe any one justifiable in using amalgam.

That this material is used by some who stand high in the profession, we do not pretend to deny, but if it can be shown, as we think it already has been, that gold can be successfully employed in those very cases, in which, *only*, they recommend the use of amalgam, condemning it as they do in all others, the opinion and example of such practitioners upon this subject is worth nothing at all. We have been referred to a few gentlemen of high professional standing by some of the advocates for the use of the article in question, as justifying their own practice with regard to it, but we cannot, with our present knowledge and experience upon the subject, consent to be influenced by their opinions in this matter, and, therefore, must be excused for dissenting from them, and adopting a practice which we conceive to be more in accordance with sound therapeutical principles.

In thus differing with gentlemen, for many of whom we entertain feelings of friendship and esteem, and whose opinions upon other subjects connected with the science and art of dental surgery, we highly respect, we are actuated by no other motive than a desire to promote what we sincerely conceive to be correct practice; and believing that the use of amalgam for filling teeth is fraught with pernicious effects, we feel bound to discountenance its employment.

But while there are some, even among those whose opinion on many subjects, we highly respect, who believe that amalgam may be, *sometimes*, advantageously employed, we are happy to know that its use, in *all cases*, is condemned by others, both in this country and Europe, who, for scientific attainments and practical skill are unsurpassed. It has, also, by unanimous vote, been pronounced by the Virginia and Mississippi Valley Associations of Dental Surgeons, to be mal-practice.

As to the chemical properties of amalgam, and the changes it is liable to undergo in the mouth, these have all been so accurately enumerated by others, that we do not deem it necessary to recapitulate them at this time. We shall, therefore, conclude our remarks, for the present, upon this subject, by simply observing, that, as for ourself, we desire no better material for filling teeth than GOLD.

Since writing the foregoing, we have received the New York Journal of Medicine for November, which contains two articles upon the use of the material under consideration—one from Dr. John Trenor, and the other from Dr. Eleazar Parmly. The first seems to be a labored effort to prove that amalgam may be advantageously employed in many cases—especially for filling the deciduous teeth, and that it cannot in any case undergo decomposition or oxydation and exert a deleterious effect upon the mouth or general system. He narrates some cases in support of the position he assumes, but most of the facts connected with them are so singularly at variance with well established principles of pathology that they can only be regarded as exceptions to the general rule. We have met with many cases where effects similar to those described by Dr. T. had been produced by amalgam fillings in *living* teeth, which were removed by replacing them with gold, but have yet to see the *first*, where they have developed themselves around *dead* teeth, and have been *completely* removed while the teeth were permitted to remain in the mouth. Now if the teeth which Dr. T. mentions did not act as *local irritants*, and if the cause, as he supposes was wholly constitutional, the gums would have been *equally* affected around all the teeth. But if, on the other hand, the teeth in question did act as *morbid irritants*, as they evidently did, the correct practice would have been to have extracted the teeth.

With regard to the chemical changes which amalgam often undergoes in the mouth, Dr. T's assumption is opposed by too many facts to be worth any thing at all. In fact, every position which he assumes is shown, by the most incontrovertible facts, adduced by Dr. Parmly, in the same number of the Journal in which his article appears, to be erroneous.

That the oxydation and discoloration of amalgam fillings may be favored by galvanic action, we think very probable. At any rate, we come to this conclusion some eight or nine years ago, and wrote a short article upon the subject, which was published at the time, in a Baltimore daily paper. This opinion is ably maintained by Dr. Slack, professor of chemistry, in the Ohio Medical College, in a well written paper copied into the present number of the Journal from the Dental Register, and by professor Westcott, as may be seen by the article of Dr. Parmly alluded to above.

Finally, assuming that the articles of Drs. Trenor and Parmly contain a fair exposition of the views of the advocates for, and opposers of the use of amalgam, together with the arguments for and against it, we should be glad to copy both into the Journal, but cannot do so at present, for want of room.—*Balt. Ed.*

